# A taxonomy for consistent handling of conditions not related to the spinal cord injury (SCI) in the International Standards for Neurological Classification of SCI (ISNCSCI)

**DOI:** 10.1038/s41393-021-00646-0

**Published:** 2021-06-09

**Authors:** Rüdiger Rupp, Christian Schuld, Fin Biering-Sørensen, Kristen Walden, Gianna Rodriguez, Steven Kirshblum, ASIA International Standards Committee, Randal Betz, Stephen P. Burns, William Donovan, Daniel E. Graves, James Guest, Linda Jones, Andrei Krassioukov, Mary Jane Mulcahey, Mary Schmidt Read, Keith Tansey

**Affiliations:** 1 Spinal Cord Injury Center, Heidelberg University Hospital, Heidelberg, Germany; 2 Department for Spinal Cord Injuries, Rigshospitalet, University of Copenhagen, Copenhagen, Denmark; 3 Praxis Spinal Cord Institute, Vancouver, Canada; 4 University of Michigan, Michigan Medicine, Ann Arbor, MI USA; 5 Kessler Institute for Rehabilitation, West Orange, NJ USA; 6 Rutgers New Jersey Medical School, Newark, NJ USA; 7 Institute for Spine and Scoliosis, Ocean City, NJ USA; 8 Department of Rehabilitation Medicine, University of Washington School of Medicine, Seattle, WA USA; 9 Spinal Cord Injury Service, VA Puget Sound Health Care System, Seattle, WA USA; 10 Institute for Rehabilitation and Research, Houston, TX USA; 11 Thomas Jefferson University, Philadelphia, PA USA; 12 University of Miami, Miller School of Medicine, Miami, FL USA; 13 International Collaboration on Repair Discovery (ICORD), University of British Columbia, Vancouver, BC Canada; 14 GF Strong Rehabilitation Center, Vancouver, BC Canada; 15 Vancouver Coastal Health Research Institute, Vancouver Coastal Health, Vancouver, BC Canada; 16 Magee Rehabilitation Hospital, Jefferson Health, Philadelphia, PA USA; 17 Departments of Neurosurgery and Neurobiology, University of Mississippi Medical Center, Jackson, MS USA; 18 Center for Neuroscience and Neurological Recovery, Methodist Rehabilitation Center, Jackson, MS USA; 19 Spinal Cord Injury Medicine and Research Services, Jackson VA Medical Center, Jackson, MS USA

**Keywords:** Outcomes research, Spinal cord diseases

## Abstract

**Study design:**

Committee consensus process including additional structured feedback from spinal cord injury (SCI) experts attending a focus group workshop.

**Objectives:**

To define a taxonomy for standardized documentation of non-SCI-related conditions in the International Standards for Neurological Classification of SCI (ISNCSCI).

**Setting:**

Americal Spinal Injury Association (ASIA) International Standards Committee with 16 international ISNCSCI experts.

**Methods:**

With the new taxonomy, not-normal sensory or motor scores should be tagged with an asterisk (“*”), if they are impacted by a non-SCI condition such as burns, casts, joint contractures, peripheral nerve injuries, amputations, pain, or generalized weakness. The non-SCI condition and instructions on how to handle the “*”-tagged scores during classification should be detailed in the comments box. While sum scores are always calculated based on examined scores, classification variables such as the neurological level of injury (NLI) or the ASIA Impairment Scale (AIS) grades are tagged with an “*”, when they have been determined on the basis of clinical assumptions.

**Results:**

With the extended “*”-tag concept, sensory and motor examination results impacted by non-SCI conditions above, at, or below the NLI can be consistently documented, scored, and classified. Feedback from workshop participants confirms agreement on its clinical relevance, logic and soundness, easiness of understanding, communicability, and applicability in daily work.

**Conclusions:**

After multiple internal revisions, a taxonomy for structured documentation of conditions superimposed on the impairments caused by the SCI together with guidelines for consistent scoring and classification was released with the 2019 ISNCSCI revision. This taxonomy is intended to increase the accuracy of ISNCSCI classifications.

## Introduction

The International Standards for Neurological Classification of Spinal Cord Injury (ISNCSCI) of the American Spinal Injury Association (ASIA) and the International Spinal Injury Society (ISCoS) represent the internationally adopted standardized clinical neurological examination and classification for individuals with spinal cord injury (SCI) [1]. The ISNCSCI is intended to determine the level and the severity of the SCI in all phases after injury [2]. The basic concept of the ISNCSCI is assessing the integrity of spinal cord function by testing the sensory functions in 28 dermatomes and the muscle functions of ten key muscles of the upper and lower extremities for both sides of the body. It represents a well-established standard for clinical documentation and communication of SCI-related neurological impairments as well as for defining inclusion/exclusion criteria, stratification, and outcome assessment in research studies [3]. In neurotherapeutic clinical trials in SCI, in particular Phase II trials, recruitment of a sufficient number of participants over a short period of time represents a huge challenge [4]. Major confounding factors of correct ISNCSCI classifications are non-SCI conditions that prevent examination of myotomes or dermatomes (e.g., limb swelling, pain, burns, casts, etc.), cause additional impairments (e.g., peripheral nerve injuries, musculoskeletal injuries ranging from transient movement restrictions up to permanent conditions such as amputations), or result in a generalized weakness (e.g., due to severe disuse, aging). Therefore, every effort should be undertaken to allow for a consistent, accurate, and precise ISNCSCI classification even where non-SCI conditions altering the examination results are present. This is especially important if ISNCSCI examination results or classifications are correlated with functional outcomes [5, 6].

In the 2003 ISNCSCI revision, the “5*” was introduced as a separate grade in the motor examination. A “5*” was recommended to be recorded when the examiner believed that the function of a myotome would be graded as normal if inhibiting factors (i.e., pain, disuse) were not present. The rationale for the asterisk or star-tag following the “5” as a grade for normal muscle strength was twofold: (1) a “5*” could easily be replaced by a “5” during classification and (2) the classification (especially the motor level determination) with the “5” should be performed as usual and did not need additional classification rules.

However, the use of the “5*” had several disadvantages: If the actual examined motor score was not documented (in the comments box), changes in muscle strength assessed in follow-up examinations could not be quantified or—even worse—would be misinterpreted as deterioration. In addition, no rules had been defined on how to correctly determine sum motor scores in the presence of one or more “5*”s. Simply replacing a “5*” with a normal motor score might result in a low correlation with functional assessments such as the Spinal Cord Independence Measure (SCIM) [7]. Similar to the motor exam, sensory scores might also be rated as normal if confounding factors were not present, although there was no option of a separate grade to denote this. Among others, examples of non-SCI conditions affecting sensory function are body areas with skin burns or pressure injuries, the presence of musculoskeletal pain, or the swelling of limbs.

The aim of this work was to develop a taxonomy in ISNCSCI for consistent and accurate documentation of all focal non-SCI-related conditions above, at, and below the Neurological Level of Injury (NLI) superimposed on the sensory and/or motor impairments caused by the SCI. With the changes of the 2019 ISNCSCI revision, consistent scoring and classification in the presence of non-SCI conditions such as peripheral nerve lesions, pain, or general weakness are now possible. This paper is intended to supplement the ISNCSCI booklet [8] and acts as a reference to support clinicians in the correct application of the non-SCI taxonomy.

## Methods

Starting in 2016, a subcommittee of the International Standards Committee of ASIA formed by the authors, who expressed their interest in the topic, started its work to develop a taxonomy for consistent documentation of non-SCI conditions. To overcome the major drawback of the “5*” score, which is the non-documentation of actual examination scores, the committee agreed to document the examined sensory or motor score including “not-testable (NT)” on the worksheet. To indicate that an examination result is believed to be impacted by a non-SCI condition, it was agreed to provide the examiner with the opportunity to tag an examination score with an asterisk (“*”) symbol. This “*”-symbol can be used to tag any abnormal or not-testable (NT) score irrespective of its segmental level (above, at, or below the NLI). If any score has been tagged with a “*”, details about the non-SCI condition and instructions on how to replace the examined score(s) with assumed score(s) during classification must be provided in the comments box. Since the main purpose of ISNCSCI is the determination of the level of the injury to the spinal cord and the severity of the neurological impairments caused by the SCI, the assumed sensory or motor scores used for later classification are those that an experienced examiner thinks are obtained without the presence of the non-SCI condition. Specifically, the examiner should document the assumed scores as “normal for classification”, as a concrete score (i.e., available from a prior exam), or as “not-normal for classification” as the least specific assumption of the impact of the non-SCI condition. In any case, the use of the comments box is of utmost importance, because without the latter information, consistent classification of cases with “*”-tagged scores is not possible. Regarding the replacement of “*”-tagged examination scores during classification, there are basically three constellations that might occur, which are described below:

1. An examiner thinks that the sensory and/or motor function would be normal if the non-SCI condition was not present. During the classification process, which consists of the determination of the sensory and motor levels, the single NLI, the ASIA Impairment Scale (AIS) grade, and the Zones of Partial Preservation (ZPPs), examined scores tagged with a “*” are considered to have normal values (assumed score for motor score = 5, assumed score for sensory score = 2). This is always the case if non-SCI conditions are present above the NLI. An example would be a fracture in the upper extremity that impacts sensory and motor examination scores in a person with a low-thoracic SCI.
2. An examiner might not be able to differentiate the SCI-related impairment from the one caused by the non-SCI condition, meaning that s/he is unable to determine the impairment solely caused by the SCI. This is the case if the non-SCI condition is present in segments caudal to the NLI or in segments at the NLI (including one or two segments rostral to it). An example would be an injury of the peroneal nerve in an individual with a high thoracic SCI. In these cases, the most conservative approach in classification would be to assume that the motor and/or sensory function is “not normal” from the perspective of the SCI-related impairment. Although the exact impairment caused by the SCI on sensory/motor function is not known, it is most likely that it will lead to an abnormal sensory/motor score below the NLI. Consequently, it needs to be checked whether classifications with the actual examination score and all other possible abnormal scores (those greater than the examined score and less than normal (= 5 for motor scores, = 2 for sensory scores)) lead to a unique result (more details below).
3. In cases of neurologically stable, chronic SCI, information about the impairment caused by the SCI alone might be available from a prior examination when the non-SCI condition was not yet present. An example would be a follow-up exam of an individual with a chronic, mid-thoracic lesion presenting with a cast (and therefore not-testable muscle functions) due to a recent fracture of the tibia. If the examiner assumes that the prior examination results reflect the SCI-related impairments, she/he should provide details on the prior examination results in the comments box together with the statement to use those for classification.

### Implications of “*”-tagged scores on motor and sensory total scoring

As a consequence of recording the actual sensory and motor scores, the total light touch (LT) and pinprick (PP) sensory scores as well as upper/lower extremity and total motor scores are calculated on the basis of the examined scores, even in the presence of non-SCI conditions (unless NT or NT* is present, then sum scores are eventually not determinable and rated as “ND”). This means that sum scores are never tagged with an “*”.

### Implications of “*”-tagged scores on classification

During the examination, the “*” is used to mark abnormal scores when they are thought to be affected by a non-SCI condition. An examiner then needs to provide additional information in the comments box, on how s/he thinks these “*”-tagged scores should be handled during classification. The assumed scores used for classification represent those an examiner thinks would be present without the non-SCI condition. This means, that the classification results in the presence of “*”-tagged scores may be dependent on clinical assumptions. To clearly indicate that a classification variable, i.e., all levels, the AIS grades, and the ZPPs, has been determined based on assumptions, it should be tagged with an “*”.

However, classification variables do not always have to be tagged in the presence of “*”-tagged examination scores. There are cases in which the classification results based on the assumed scores and those using the actual examined scores are the same. In these cases, the resulting classification variables do not need to be tagged with an “*”. In some complex cases, it might be challenging to determine whether a classification variable such as the sensory/motor levels, the NLI, the AIS grade or the ZPPs should be tagged with an “*” or not. To support examiners in correctly tagging classification variables, a standardized workflow has been recommended:

1. Perform the classification replacing the “*”-tagged scores with the assumed scores.
2. Record the classification results based on the assumed scores (which include the examiner’s judgment) in the respective classification boxes at the bottom of the worksheet.
3. Next, re-classify the case with the examined scores (while ignoring the “*”-tags).
4. Tag each classification variable which is different between classification with the assumed scores and re-classification with the examined scores with an “*” to indicate that it is based on clinical assumptions.

During the classification of cases, where the non-SCI impairment can be clearly discriminated from the SCI-related impairment (mostly if above the NLI), the tagged examined scores should be replaced for classification by the scores assumed by the examiner (5 for motor score and 2 for sensory score if clearly above the NLI) to be present without the non-SCI condition.

In cases where the non-SCI impairment cannot be isolated from the SCI-related impairment (around or below the NLI), the most conservative approach is to assume that the sensory and motor functions at and below the NLI are impaired due to the SCI. Therefore, an examiner will likely assume the “*”-tagged scores to be not normal if the non-SCI condition would not be present. Consequently, because the impact of the non-SCI condition on the sensory and motor function is unknown, in most cases not a single score can be assumed for classification, but the examined score and all other scores greater than the examined score, except normal. In such cases, the classification according to the above workflow is first performed based on the examined scores and subsequently for all other possible scores greater than the examined score except normal. If more than one score is “*”-tagged, the aforementioned classification procedure has to be performed for every tagged score. If the classifications with all combinations of examined and assumed scores do not converge to a single, unique result for a classification variable, “ND*” is recorded for this variable. Otherwise the unique (untagged) result is documented. Due to the fact that the number of combinations of examined and assumed scores grow exponentially with the number of tagged scores, the determination of correct classification results becomes more challenging in such cases. Here, computer algorithms might effectively support examiners in correct classification. The underlying classification principle in these cases follows the one for not-testable segments where multiple classifications with all possible combinations of assumed scores are performed and checked if they converge in a unique classification result [9].

Besides the general procedures for classification in the presence of “*”-tagged scores due to non-SCI conditions, specific rules have been compiled for each classification variables, which are described in the following paragraphs.

### Sensory/motor levels and NLI

If sensory/motor levels or the NLI are impacted by “*”-tagged segmental scores, then these levels should also be marked with an “*”. This holds true for all cases with “*”-tagged scores above-level or at-level. The NLI needs to be tagged if any of the most rostral levels of all sensory and motor levels is tagged with an “*”.

### AIS grade

In the determination of the AIS grade, the use of “*”s might have an impact on AIS classification in some cases and in others not. For example, an AIS A classification without a non-SCI condition such as a skin burn or pressure injury present in the lowest sacral segments and therefore no tagged sensory scores (LT and PP sensation in S4–5 including deep anal pressure sensation (DAP)) and no non-SCI condition present that leads to a tagged voluntary anal contraction (VAC), is never marked with an asterisk. In cases where the replacement of tagged examination scores with assumed ones has an impact on the AIS grade, it should be tagged with an asterisk.

### Complete vs. incomplete

In cases with no sacral sparing of any sensory or motor function or in whom the sacral examination cannot be performed (one or more “NT”s in LT or PP in S4–5, VAC or DAP), but the examiner thinks that sensory or motor function would be preserved in the lowest sacral segments without the non-SCI condition present, “Incomplete*” would be recorded. The “*” indicates that this classification is based on clinical assumptions. If the motor or sensory examination of the lowest sacral segments cannot be performed due to a non-SCI condition such as a pressure injury or skin burn, but the examiner thinks that no sacral sparing of any sensory or motor function is preserved, this would result in a “Complete*” classification. In all other cases, the “Complete/Incomplete” classification is not tagged with an asterisk.

### ZPP

The 2019 ISNCSCI revision introduced an expanded definition of the ZPP to include some incomplete injuries. The sensory ZPP for one side of the body is now defined in all cases with missing DAP and absent sensory function (LT and PP sensation) in the lowest sacral segments on this side. The motor ZPP is defined in all cases with missing VAC. In case there is no sensory or motor function preserved below an “*”-tagged sensory or motor level on a given side, the “*”-tagged sensory or motor level should be recorded in the respective ZPP box as the most caudal extend of sensory/motor function on this side. In case sensory or motor function is preserved below the sensory or motor level, the sensory or motor ZPPs do not need to be tagged with an “*”, when the most caudal segments with preserved sensory or motor function do not contain “*”-tagged scores. However, a sensory or motor ZPP should be tagged with an “*”, indicating that it is based on clinical assumptions if a “0*” or “NT*” is present in a segment with totally absent sensory or motor function caudal to it and an examiner thinks that function would be preserved in this “*”-tagged segment, if the non-SCI condition was not present. In this case, the “0*” or the “NT*” is replaced by a score greater than 0 during classification resulting in an “*”-tagged ZPP level at the level of the “0*” or “NT*”.

The first draft of this non-SCI taxonomy was compiled in February 2018 and discussed among the members of the subcommittee. The final draft was presented in a 20-min talk as part of a scientific ISNCSCI workshop at ASIA’s annual meeting in Rochester, MN (USA) in May 2018 [10]. After this workshop, structured feedback was collected from the participants via a questionnaire. Participants were asked to answer five multiple-choice questions, including information on their profession, self-rated experience in SCI medicine, in the ISNCSCI exam and classification, and their frequency of regularly applying ISNCSCI. In the second part of the questionnaire, participants rated their level of agreement with seven statements about the non-SCI taxonomy on a 5-point Likert Scale (1 = strongly agree, 5 = strongly disagree). These statements included clinical relevance, logical- and soundness, ease of understanding and teachability, the importance for clinical routine and research, communicability, applicability in the own daily work, and the daily work of others. At the end of the questionnaire, people could give comments in the free-text form.

## Results

A total of 15 participants provided feedback on the non-SCI taxonomy during the workshop at the 2018 ASIA meeting. This focus group consisted mainly of physicians with more than 10 years of experience in SCI medicine, rated themselves as highly experienced in performing both ISNCSCI exams and classifications, with the majority using ISNCSCI more than once a week (details in Table 1).

**Table 1:** Characteristics of the workshop attendees providing feedback on the non-SCI taxonomy.

Survey item	Results
Profession	9 physicians, 2 physiotherapists, 2 researchers, 1 other
Years of experience in SCI medicine	1 with less than 1 year, 1 with 1–5 years, 2 with 6–10 years, 11 with >10 years
Self-rated experience in ISNCSCI exam	1 novice, 3 experienced, 8 highly experienced, 3 experts
Self-rated experience in ISNCSCI classification	1 novice, 3 experienced, 7 highly experienced, 4 experts
Frequency of ISNCSCI exams/classifications	3 less often than 1× a month, 3 once a month, 5 once a week, 4 once a day

*SCI* Spinal Cord Injury, *ISNCSCI* International Standards for Neurological Classification of SCI.

The 15 respondents rated all statements between “strongly agree [1]” and “agree [2]” with the highest agreement (mean 1.5) with the statement that the taxonomy addresses a clinically relevant topic and the lowest agreement (mean 2.3) with the ease to teach (Table 2). There were free-text comments provided by the participants, however, they did not contain substantial concerns.

**Table 2:** Content and results of the survey handed over to the participants of the focus group workshop at the 2018 American Spinal Injury Association meeting.

What is your position concerning the following statements:	Strongly agree (1)	Agree (2)	Neither agree nor disagree (3)	Disagree (4)	Strongly disagree (5)	Mean
The proposed taxonomy for documentation of non-SCI conditions addresses a clinically relevant topic	10	3	2	0	0	1.5
The proposed non-SCI taxonomy is logical and sound	6	7	2	0	0	1.7
The proposed documentation scheme of non-SCI conditions is easy to understand	5	7	3	0	0	1.7
The proposed non-SCI taxonomy is important in clinical routine	5	8	1	1	0	1.9
The proposed non-SCI taxonomy is important for research	7	6	1	1	0	1.7
The proposed non-SCI taxonomy is easy to teach	3	6	5	1	0	2.3
I will use the proposed non-SCI taxonomy in my daily work	4	10	0	1	0	1.9
I think that others will use the proposed non-SCI taxonomy in their daily work	4	8	2	1	0	2.0

*SCI* Spinal Cord Injury.

The numbers below the rating categories from “Strongly agree (1)” to “Strongly disagree (5)” represent the number of replies in each of the categories.

After a total of 11 revisions, a consensus on the non-SCI taxonomy was reached among the members of the subcommittee in October 2018. Following the policies and procedures for revisions to the ISNCSCI [11], the proposed changes were first approved by all members of the ASIA International Standards Committee at the end of January 2019 and in February 2019 by the board of ASIA and ISCoS representatives. The non-SCI taxonomy was then integrated into the 8th edition of the ISNCSCI booklet [8] released in April 2019 and later in November 2019 into the International Standards Training e-Program (InSTeP) [12].

For an illustration of the application of the new ISNCSCI non-SCI taxonomy, three example cases assessed and classified according to the seventh (2015, [13]) (subfigure A in each example) and eighth ISNCSCI (2019, [8]) (subfigure B) editions are presented:

### Example case 1: thoracic SCI with concomitant partial injury of the brachial plexus

**Fig. 1:**
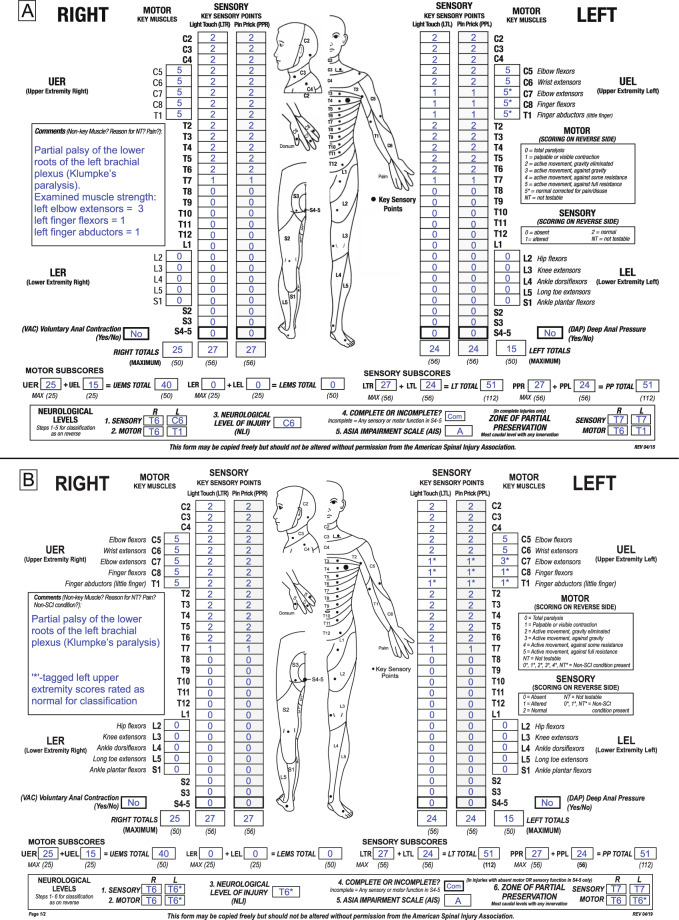
Sample case 1 of an individual with an injury of the thoracic spinal cord and pre-existing partial lesion of peripheral left upper extremity nerves. **A** Classification according to the 2015 International Standards for Neurological Classification of Spinal Cord Injury (ISNCSCI) edition. **B** Classification according to the 2019 ISNCSCI edition.

An example for an above-level non-SCI condition would be an individual with paraplegia due to an SCI at T6 and a concomitant partial brachial plexus lesion (Fig. 1). The peripheral nerve lesion causes impairments in the dermatomes and myotomes innervated by the spinal segments C7, C8, and T1. This case will be scored, classified, and discussed twice: in Fig. 1A using the ISNCSCI revision updated in 2015 and in Fig. 1B using the current ISNCSCI 2019 revision.

Following the 2015 ISNCSCI rules, an examiner would rate the motor scores of the myotomes C7, C8, and T1 as “5*” and provide the reason for it in the comments box (“Partial palsy of the lower roots of the left brachial plexus (Klumpke’s paralysis)”). Although not explicitly recommended in the 2015 booklet, good practice implied to also document the real examination scores in the comments box (Fig. 1A). The left sensory level and thus the NLI would be classified as C6, because LT and PP sensation are normal to C6 with altered sensory function in C7. This is obviously not in line with the clinical judgment that the SCI is in the thoracic region. In the 2015 ISNCSCI version, no guidelines were provided on how to correctly calculate sum motor scores in the presence of “5*” grades, which left room for interpretation. In Fig. 1A, the sum motor scores were calculated on the basis of the real examination values, however, an examiner might have used another option such as replacing a “5*” with a “5” during summation. This case is classified as AIS A due to missing sacral functions. This AIS A classification is not influenced by the presence of the non-SCI condition of the left arm.

According to the 2019 ISNCSCI revision, the same case (Fig. 1B) is scored and classified as follows: For the segments C7 to T1, the actual sensory and motor examination scores are recorded in the respective boxes. Sum scores are calculated on the basis of the actual examination scores and never tagged. Each sensory and motor score of the segments C7, C8, and T1 is tagged with an “*” to indicate the presence of the non-SCI condition. The reason for the “*”-tagged scores is given in the comments box (“Partial palsy of the lower roots of the left brachial plexus (Klumpke’s paralysis)”) together with the judgment of the examiner that the “*”-tagged scores should be handled as normal during classification. Replacing the “*”-tagged scores with normal scores leads to T6* as sensory and motor levels on the left side. All levels on the left side as well as the NLI are tagged with a ‘*’ to indicate that they have been determined based on clinical assumptions. In respect to the AIS, grade A does not need to be tagged, because it is in this case not affected by the non-SCI condition. The left motor ZPP equals the left motor level of T6*, because no motor function is preserved below T6 (Reminder: unlike the motor level which does follow the sensory level, the motor ZPP does not follow the sensory ZPP in regions with no myotomes to test such as the thoracic region). The left motor ZPP needs to be tagged with an “*” because it follows in this case the motor level, which has been determined based on clinical assumptions. The sensory ZPPs of both sides (all T7) and the right motor ZPP (T6) are determined as usual.

### Example case 2: cervical SCI with pre-existing partial injury of the peroneal nerve

**Fig. 2:**
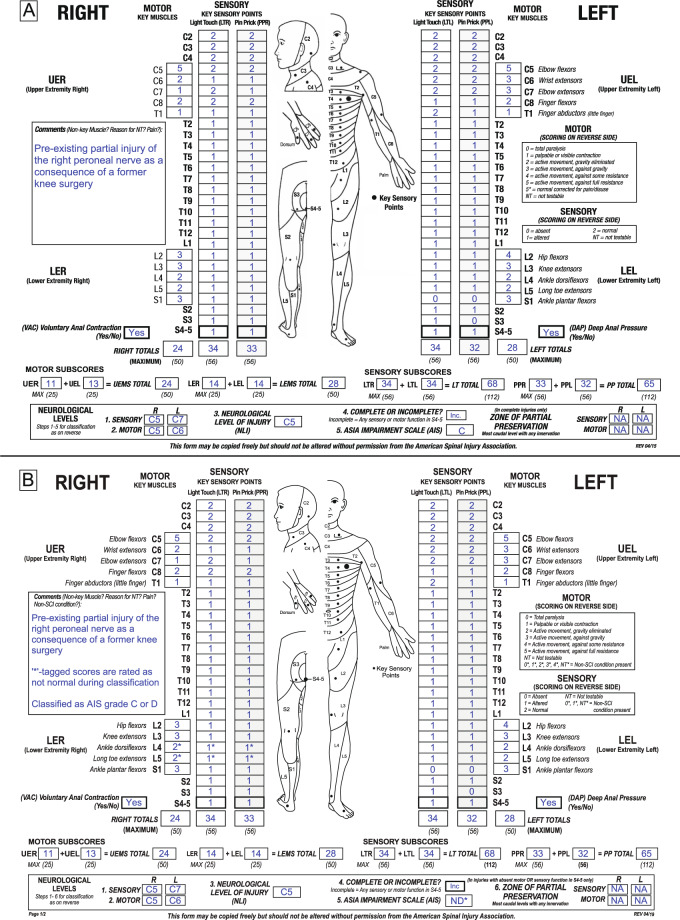
Sample case 2 of an individual with an injury of the cervical spinal cord and pre-existing partial lesion of the right peroneal nerve. **A** Classification according to the 2015 International Standards for Neurological Classification of Spinal Cord Injury (ISNCSCI) edition. **B** Classification according to the 2019 ISNCSCI edition.

An example of a below-level non-SCI condition would be an individual with tetraplegia due to an SCI at C5 and a pre-existing injury of the right peroneal nerve (Fig. 2). The peripheral nerve lesion causes impairments in the dermatomes and myotomes innervated by the spinal segments L4 and L5. Following the 2015 ISNCSCI rules, an examiner would document the actual examination scores of these segments and perform the classification as usual (Fig. 2A). The examiner would not use a “5*” as motor score because the impact of the non-SCI condition on the examination results cannot be judged and a normal muscle function without the presence of the non-SCI condition can never be assumed below the level of injury. This individual would be classified as AIS C, because less than half of the key muscles below the NLI of C5 (8 out of 18) have a grade greater or equal to 3.

According to the 2019 ISNCSCI revision, this case is classified as follows (Fig. 2B): The sensory and motor scores of L4 and L5 are tagged with a “*” to indicate the presence of the non-SCI condition. The non-SCI condition is then explained in the comments box (“Pre-existing partial injury of the right peroneal nerve as a consequence of a former knee surgery”). In addition, information is provided that these scores should be treated as not normal for classification purposes because the impairments caused by the non-SCI condition are superimposed to those originating from the SCI. Sum scores are calculated on the basis of the examined scores and are not tagged. Due to the fact that the NLI is well above the non-SCI condition, levels can be uniquely determined even though the non-SCI condition is present.

However, for determination of the AIS grade all not-normal motor scores (actual examined motor scores and all other abnormal scores greater than the examined score (up to “4”) in L4 and L5 need to be considered. This results in scenarios, in which this case is classified as AIS C (motor scores in L4 and L5 equal to 2) or AIS D (motor scores in L4 or L5 greater than 2). Therefore, taking clinical assumptions into account results in a non-determinable AIS grade and should be noted as “ND*”. However, it should be noted in the comments box that this case is classified as motor incomplete, i.e., AIS C or D.

### Example case 3: chronic cervical SCI with elbow cast

**Fig. 3:**
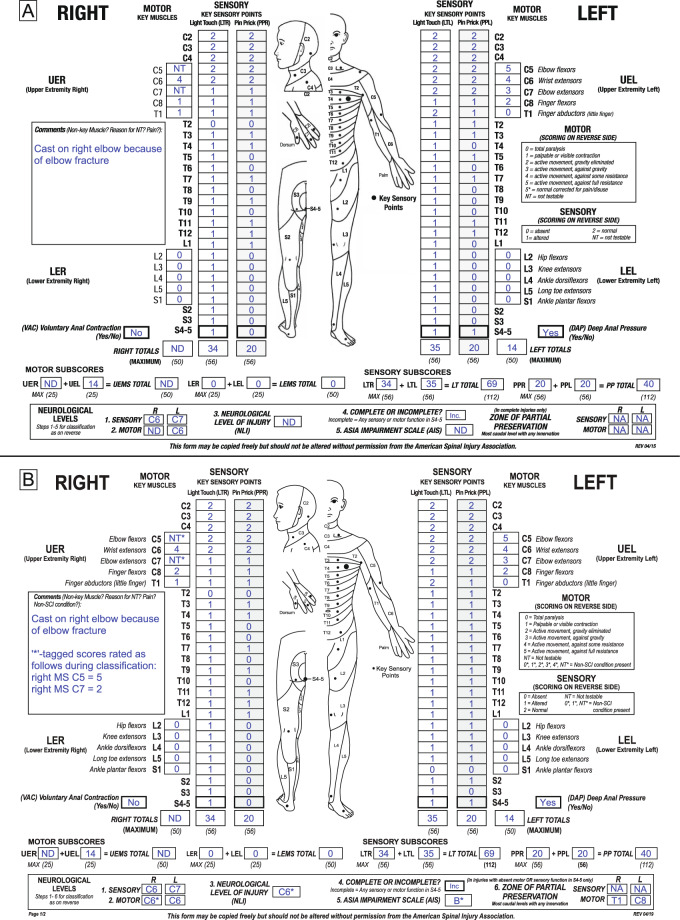
Sample case 3 of an individual with an injury of the cervical spinal cord and a cast at the right elbow. **A** Classification according to the 2015 International Standards for Neurological Classification of Spinal Cord Injury (ISNCSCI) edition. **B** Classification according to the 2019 ISNCSCI edition.

An example for an at-level non-SCI condition would be an individual with chronic tetraplegia due to an SCI at C5 and a cast on the right elbow due to a recent fracture (Fig. 3). While alternative points within the dermatomes C5 and T1 for testing LT and PP sensation are accessible in this individual, the cast does not allow for motor testing of elbow flexors (C5) and extensors (C7). Following the 2015 ISNCSCI rules, an examiner would document the motor score of C5 and C7 on the right as “NT” (Fig. 3A), because the muscles cannot be tested due to the cast. The reason for the “NT”s is given in the comments box. The upper extremity motor score and the total motor score cannot be determined because of the non-testable elbow muscles. The motor level on the right cannot be uniquely determined (recorded as “ND”): A normal motor function in C5 on the right leads to a right motor level of C6, while an abnormal function results in a motor level of C4. Due to the preservation of sensory function in S4–5, this case is classified as AIS B in the case of a right motor level of C6. However, in the case of a right motor level of C4 and thus motor function preserved more than three segments below the motor level, the patient is classified as AIS C. This leads to a non-determinable (“ND”) AIS grade.

According to the 2019 ISNCSCI revision, this case is classified as follows (Fig. 3B): The not-testable right elbow flexors and extensors are graded as “NT*” and the reason is given in the comments box. In this case, results from a prior exam (motor score C5 = 5; motor score C7 = 2) are available and should be used during classification. With this information given in the comments box, the motor level on the right and the NLI are classified as C6*, because they are based on the assumption that the C5 myotome is graded as normal. When using the motor scores from the prior exam, this case is classified as AIS B*. The AIS grade has to be tagged, because without the clinical assumption of a normal right C5 myotome, this case would be classified as AIS C with motor functions preserved more than three segments below the right motor level of C4 or C5. The sensory ZPPs are not applicable because of preserved sensory functions in S4–5. The caudal extent of the motor ZPPs can be determined as usual (right motor ZPP = T1, left motor ZPP = C8).

## Discussion

The question of how to allow for conclusive integration of clinical judgment in the presence of non-SCI-related impairments has been a subject in ISNCSCI editions for many years [14]. In the 2003 reference, the “5*” was introduced as a dedicated motor score to provide the examiner with the opportunity to indicate that an abnormal motor score would be graded as normal if the inhibiting non-SCI condition is not present [15]. However, with the 2019 ISNCSCI revision a taxonomy based on a generalized use of the asterisk has been introduced that allows a systematic documentation of focal non-SCI-related conditions impacting the sensory and/or motor examination results. In addition, guidelines for handling of “*”-tagged scores caused by non-SCI conditions during scoring were implemented. The committee devoted special attention to consistently integrate the tagging concept into the scoring as well as the classification scheme. A general concept extended by specific rules for tagging of each classification variable has been introduced.

In response to a call for difficult ISNCSCI cases published in ASIA’s newsletter in January 2019, 15 cases were sent in, from which 8 were related to the presence of non-SCI conditions. This is in line with the feedback given by the 2018 ASIA workshop participants that consistent handling of non-SCI conditions is relevant for clinical as well as research purposes. Only with the new non-SCI taxonomy, all eight difficult cases could be comprehensively scored and consistently, transparently, and precisely classified.

A qualitative analysis of ISNCSCI datasets collected in 5.474 individuals with SCI between 2001 and 2019 in the registry of the European Multicenter Study about Spinal Cord Injury (EMSCI) revealed that in 128 individuals (2.3%) in the first year after trauma at least one myotome was rated as “5*” indicating the presence of a non-SCI condition. Looking at the numbers from different centers, the range of the percentage of individuals with “5*” motor scores differed substantially from 0 to 18%. In 21 of the 33 centers, no ISNCSCI dataset contained a “5*”. We can only speculate about the reasons for this. Personnel of all EMSCI centers were trained in the ISNCSCI examination and classification since 2006 and should therefore be familiar with this muscle grade and its concept [16]. A possible explanation for the non-use of the “5*” could be that due to the lack of further guidelines for scoring and classification, centers found it too complicated and were consequently excluding cases with non-SCI conditions present. All these numbers show that although the non-SCI taxonomy will only be used in a small number of exams, it could have a large impact on ensuring correct classification in those cases.

We see a potential risk that the new non-SCI taxonomy contributes to the complexity of ISNCSCI. Although the committee undertook every effort, e.g., use of only one tag symbol (the “*”) universally applicable above, at, and below the level of lesion, to keep the taxonomy as simple as possible, examiners might find it hard to fully understand the concept. This is supported by the fact that in the survey of the 2018 ASIA workshop, highly experienced attendees rated the teachability of the taxonomy as only moderate. As a result, the proper application of the non-SCI taxonomy needs training, and sufficient time should be spent in instructional courses on the teaching of the correct use of the “*”-tag, its implications on classification, and on discussing a number of sample cases with non-SCI conditions present. In this respect, ISNCSCI computer algorithm implementations might serve as valuable training tools to verify manual classifications [9, 17]. Both validated ISNCSCI computer algorithms from the Praxis Spinal Cord Institute (formally Rick Hansen Institute, accessible at http://www.isncscialgorithm.com) and EMSCI (accessible at http://ais.emsci.org) are currently updated to provide the opportunity to “*”-tag examination results and handle them correctly during scoring and classification. However, this update involves several challenging aspects, such as the extraction of the numerical values of the assumed scores from the free-text entries in the comment’s box, the implementation of logic to handle “*”-tagged scores (which extends the already existing NT inference logic [9]), and finally the validation of the algorithm based on a substantial number of test cases.

With the 2019 ISNCSCI revision, a new taxonomy for accurate documentation of conditions not related to the SCI together with guidelines and rules for consistent scoring and classification in the presence of these conditions has been introduced. It is expected that with this taxonomy the quality of ISNCSCI data will further increase. In clinical trials, the taxonomy allows for standardized documentation of people who would have otherwise to be excluded from participation. By this, it contributes to inclusive study protocols.

The new non-SCI taxonomy allows for incorporating examiners’ assumptions which, however, might not always be accurate. For evaluation of inaccuracies of examiner’s assumptions, we plan to analyze data from registries that are based on subsequent ISNCSCI examinations of the same individual after acute SCI such as EMSCI or the Canadian Rick Hansen SCI Registry (RHSCIR). By comparison of the examination scores from different time points, we will be able to analyze changes in “*”-tagging of scores between examinations over time. This comparison will provide insights not only about how often examiners make corrections of their clinical judgments but also about the characteristics of the non-SCI conditions. In addition, the integration of cases with non-SCI conditions in the post-tests of ISNCSCI training courses will help to identify the challenges examiners face in their classification [16, 18].

Although the focus group providing initial feedback on the non-SCI taxonomy was small and workshop participants with less ISNCSCI experience were underrepresented, the survey results support its relevance for clinical and research purposes and its usefulness for correct classification of challenging case scenarios. Most likely, the non-SCI taxonomy adds complexity to the classification rules, and therefore training on its correct use will be needed. In registries such as from the Model SCI System (MSCIS), the RHSCIR, or from EMSCI, the consequent use of the extended “*”-concept will allow for systematic quantitative analysis of the incidence, reasons, and patterns of non-SCI conditions. Ultimately, with such data collections, the impact of the new non-SCI taxonomy on the psychometric properties of the ISNCSCI instrument and on the correlation with functional outcomes can be evaluated in the future.

## Supplementary information

Supplementary Table 1
