# Supplementary material for: A taxonomy for consistent handling of conditions not related to the spinal cord injury (SCI) in the International Standards for Neurological Classification of SCI (ISNCSCI)
Source: Spinal Cord. 2021 Jun 9;60(1):18–29. doi: 10.1038/s41393-021-00646-0 (PMC8737332; doi:10.1038/s41393-021-00646-0)
Supplement: Supplementary file 1 — Supplementary Table 1 [file 41393_2021_646_MOESM1_ESM.pdf]

R. Rupp, C. Schuld, F. Biering-Sørensen, K. Walden, G. Rodriguez, S. Kirshblum, ASIA International Standards Committee, A taxonomy for consistent handling of conditions not related to the spinal cord injury (SCI) in the International Standards for Neurological Classification of SCI (ISNCSCI), *Spinal Cord*, 2021

Supplementary Table 1. List of abbreviations

Abbreviation	Expression
ASIA	American Spinal Injury Association
AIS	ASIA Impairment Scale
DAP	Deep Anal Pressure
EMSCI	European Multicenter Study about Spinal Cord Injury
InSTeP	International Standards Training e-Program
ISCoS	International Spinal Cord Society
ISNCSCI	International Standards for Neurological Classification of Spinal Cord Injury
LT	Light Touch
MSCIS	Model Spinal Cord Injury System
ND	Not Determinable
NLI	Neurological Level of Injury
NT	Not Testable
PP	Pin Prick
RHSCIR	Rick Hansen Spinal Cord Injury Registry
SCI	Spinal Cord Injury
SCIM	Spinal Cord Independence Measure
VAC	Voluntary Anal Contraction
ZPP	Zone of Partial Preservation
